# Quadrature Frequency-Group Radar and its center estimation algorithms for small Vibrational Displacement

**DOI:** 10.1038/s41598-019-43205-7

**Published:** 2019-05-01

**Authors:** Dong Kyoo Kim, Youjin Kim

**Affiliations:** Electronics and Telecommunications Research Institute, Hyper-connected Research Lab, Daejeon, 34129 South Korea

**Keywords:** Electrical and electronic engineering, Biomedical engineering

## Abstract

The quadrature continuous-wave (QCW) radar has been extensively studied for small vibrational displacement detection such as non-contact sensing of human vital signals. One of the challenges of the QCW radar is the IQ-imbalance and DC-offset estimation by using curve fitting algorithms. Many algorithms have been proposed and have shown that the fitting error increases when the displacement length is small, in which case sufficient data is not provided to the algorithms. This paper presents a quadrature frequency-group (QFG) radar which utilizes a group of frequencies to enhance the fitting performance even with the small displacement. The grouped-frequencies in the QFG radar gives more data than the single-tone of the QCW radar under the same displacement condition. This paper presents the framework and properties of the QFG radar. Some fitting algorithms for the QFG radar are presented and the most adequate algorithm is suggested by simulation and experiments. Simulation and experimental results shows that the QFG radar outperforms the QCW radar. Specifically, it is shown that the fitting accuracy of the QFG radar is up to 100 times better than the QCW radar in the experiment.

## Introduction

**Figure 1:**
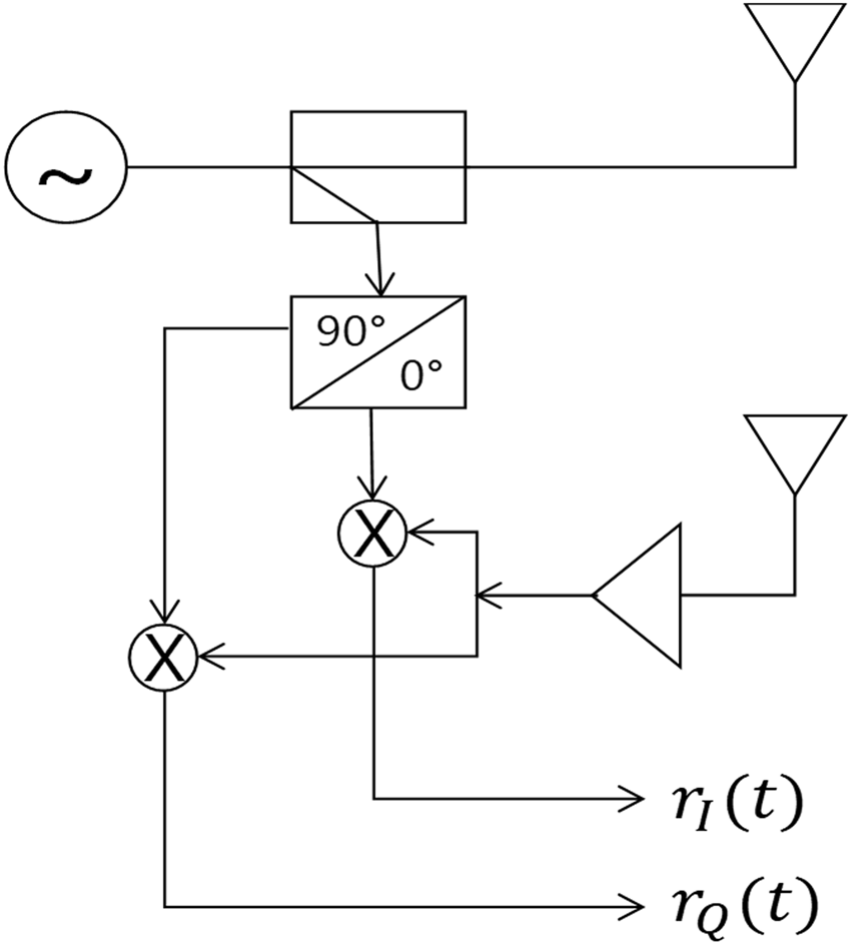
The QCW radar architecture.

Small displacement measurement using a micro-Doppler radar such a non-contact vital signal sensing has been extensively studied. Respiration, heartbeat rate, and heart rate variability are the parameters of most interest to researchers. Respiration sensing and apnea analysis^1–3^ have been widely researched, and related off-the-shelf products have been released. In particular, heartbeat signals have been intensively investigated^4–10^ where continuous wave (CW) radars have been used to detect displacement smaller than 1 *mm* on the human chest. The CW radar is a simple single transmitter and receiver with a single oscillator, in which the receiver coherently mixes the received signal down to the baseband by the oscillator. The CW radar has the so-called null point detection problem in which the baseband signal becomes null. This happens when the distance between the radar and a target is a multiple of *π*. Some literature using single mixer receiver researched to solve this problem^11–14^. Common point of the literature is that the null problem can be solved by using a phase tuning of the received and reference signal. The lack of these methods is how the receiver always reach the optimum, which is still under studying. Another one is that tunable RF phase shifting device is currently expensive and difficult to control in practice at high frequency band. Another method to solve the null problem is by using quadrature mixer receiver architecture^9^, in which the receiver has two orthogonal mix-down branches as shown in Fig. 1, where the two baseband signals are called in-phase (I) and quadrature-phase (Q) signals. This type of radar is called the quadrature continuous-wave (QCW) radar. To get useful information from direct-current (DC) of the baseband, the receiver is assumed to have the DC-coupled structure in this paper.

Small displacement induces the phase change of the baseband signal of the QCW radar, and it can be detected by linear or non-linear demodulation method^4^. Linear demodulation method finds it by assuming that it is linear to the baseband signal itself; this is valid when the displacement is much smaller than the oscillator wavelength such as displacement <*λ*/4*π*^4^. Differently from linear demodulation, non-linear demodulation gets the small displacement by calculating arctangent function of the baseband signal as *arctan*(*Q*/*I*) which gives accurate measurement of the displacement. Unfortunately the accuracy cannot be maintained unless the impairment from the QCW radar is compensated, more specifically from the radar itself and its environment. As many research articles addressed, the impaired baseband signal of the QCW radar has I/Q imbalance and DC-offset^4,15–17^. I/Q imbalance is the mismatch of magnitude or delay between the I and Q signal paths. As described in Yan *et al*.^9^ I/Q imbalance introduces errors in the arctangent function, thus it deteriorates the radar accuracy. To measure it, a phase shifter^18^ or a mechanical moving stage^19^ makes sufficient Doppler shifted signals and the baseband I and Q signals are compared. The measured imbalance can be eliminated simply by using Gram-Schmidt procedure 21. The DC-offset is the bias offset of the baseband signal, where the offsets of I and Q signals are usually different. The DC-offset also affects significant performance degradation of the QCW radar^4,17,20,21^. It is caused by hardware system imperfections and/or environment effect in-between the radar and a target^4^. If the sufficient Doppler shift is made, the baseband signal makes elliptic arc in the complex plane of the signal. By estimating the center of the ellipse, the DC-offset can be determined^16^. Several center estimation algorithms have been studied to measure the DC-offset^5,16,17,22,23^. One heuristic method using I/Q-rotation was presented in Park *et al*.^16^, however it is computationally complex but the performance is poor. The enhanced fitting algorithm was used for the center estimation in Zakrzewski *et al*.^5^, where the Levenberg-Marquardt (LM) algorithm, a positive definite geometric fitting method, is used and its performance was compared with the Park’s algorithm. Algebraic fitting method for ${\ell }_{1}$-minimization was proposed for the center estimation^17,22,23^, where its performance is better than the existing ${\ell }_{2}$-based minimization algorithms against noisy data. However, the disadvantage of the ${\ell }_{1}$-minimization is that the computationally-high linear programming is required and its real-time implementation is still be open problem.

Common point in the literature is that the center estimation performance increases when the elliptic arc is sufficiently long. To secure such a long arc, a pre-calibration step is preferred for radar systems. Usually, the pre-calibration step is performed using the calibration environment, where a metallic sphere object is placed on a mechanical moving stage. And a radar system is arranged in a straight line with the object i.e. the target. Then by controlling the stage, sufficiently long arc is generated. However, this pre-calibration fails when the center of the arc frequently changes. Actually, it changes as time goes or as the environment around the radar system changes. When the radar measures small displacement smaller than 1 *mm* on the human chest, it is difficult to secure such long arc. Gao *et al*.^20^ presented a center estimation error reduction method by correcting the estimated radius of the short arc. However, this work still requires the cumbersome calibration environment, in which the estimation data set of long arc is necessary to the radius correction of the short arc. Huang *et al*.^17^ presented a semi-definite programming algorithm for center estimation of the short arc. It requires no calibration environment, and the center estimation is performed using only the target’s small displacement. The estimation performance is comparable to the other algorithms using the calibration environment. Disadvantage of the semi-definite programming is high computational complexity, where the worst case complexity is bounded to *O*(*n*^6^). Another approach to make sufficiently long elliptic arc without pre-calibration step is proposed^24,25^. In the literature, RF phase is controlled by shifting antenna mechanically^24^ or by using RF transmitter phase shifter^25^, where additional devices are required to the radar such as machine stages^24^ and RF phase shifters^25^. At high frequency bands over 10 GHz, the high-precision machine stages and high-frequency phase shifters are currently expensive making them difficult to apply in the radar.

The previous works are all based on the center estimation of a single arc because the QCW radar uses a single-tone frequency. In this paper, we discuss the center estimation based on multiple arcs and propose the quadrature frequency-group (QFG) radar in which multiple discrete arcs are generated by a group of frequencies. The discrete arcs of the QFG radar constitute a sufficiently long elliptic arc even with the small displacement. Thus, the QFG radar requires no calibration environment, and it can be used directly in the measurement. The QFG radar requires no additional devices such as stages or RF phase shifters. Properties of the discrete arcs such as the arc length and interval are presented in terms of the frequency group. The performance improvement of the QFG radar is presented through several computer simulations such as for different carrier frequency bands, different *SNR*s, and different fitting algorithms. With the QFG radar, the existing ${\ell }_{2}$-based minimization algorithms show quite well performance, where the performance of geometric and algebraic fitting algorithms are presented. Experiments are performed in a quantitative test setup for the 24 *GHz* short-range radar system. In the test setup, the radar is closely located to a target, which is a common occurrence during human vital signal measurement. Low transmit power is assumed because of electromagnetic wave influence against human body, and the target has only small displacement such as 0.5 *mm*. The experimental results show that the fitting accuracy of the QFG radar is up to 100 times better than the QCW radar.

## Methods

### Micro-Doppler Monitoring of Small Displacement

#### Principle of the QCW radar

The QCW radar shown in Fig. 1 transmits a stable wave energy with frequency *f*, which is expressed as

1 $$s(t)={A}_{tx}\,\cos \,(2\pi ft+\varphi (t)),$$

where *ϕ* is the random phase noise of the transmitter. The transmitted signal is reflected from a target within the radar radiation area. The received baseband signal shown in Fig. 1 is written as

2 $$\begin{array}{rcl}r(t) & = & {A}_{0}\,\cos \,(4\pi {\lambda }^{-1}d+4\pi {\lambda }^{-1}x(t)+{\phi }_{0}+{\rm{\Delta }}\varphi (t))\\  &  & +\,j{A}_{0}{A}_{e}\,\sin \,(4\pi {\lambda }^{-1}d+4\pi {\lambda }^{-1}x(t)+{\phi }_{0}+{\rm{\Delta }}\varphi (t)+{\varphi }_{e})\\  &  & +\,D{C}_{I}(t)+jD{C}_{Q}(t)+w(t),\end{array}$$

where is *A*_0_ the baseband amplitude, *λ* is the wavelength of the transmitter frequency *f*, *d* is the nominal distance between the radar and a target (human chest in this paper), *x*(*t*) is the small displacement of the target (|*x*(*t*)| < *λ*), *φ*_0_ is the initial phase offset, Δ*ϕ* is the phase noise difference between the phase noise and the time-delayed phase noise, and *w*(*t*) is white Gaussian noise. The Gaussian noise variance *σ* is written as $\sqrt{{A}_{0}^{2}/(2\cdot SNR)}$. *A*_*e*_ and *ϕ*_*e*_ are amplitude and phase imbalance of the in- and quadrature-phase paths, which is mainly caused by circuit imperfection factors of the radar. Generally, the IQ-imbalance is measured at controllable test environment and corrected by the Gram–Schmidt procedure^15^

3 $$\begin{array}{rcl}[\begin{array}{c}{r}_{c,I}(t)\\ {r}_{c,Q}(t)\end{array}] & = & [\begin{array}{cc}1 & 0\\ -\tan \,{\varphi }_{e} & \frac{1}{{A}_{e}\,\cos \,{\varphi }_{e}}\end{array}]\,[\begin{array}{c}{r}_{I}(t)\\ {r}_{Q}(t)\end{array}]\\  & = & {A}_{0}\,\cos \,(4\pi {\lambda }^{-1}d+4\pi {\lambda }^{-1}x(t)+{\phi }_{0}+{\rm{\Delta }}\varphi (t))\\  &  & +\,j{A}_{0}\,\sin \,(4\pi {\lambda }^{-1}d+4\pi {\lambda }^{-1}x(t)+{\phi }_{0}+{\rm{\Delta }}\varphi (t))\\  &  & +\,D{C}_{I}(t)+jD{C}_{Q}(t)+w(t).\end{array}$$

*DC*_*I*_ and *DC*_*Q*_ in (2) are the DC-offsets of the in- and quadrature-phase paths. The DC-offset is known to be caused by reflections from target’s position as well as hardware imperfections^4^. The DC-offset induced by hardware imperfection can be easily eliminated by the pre-measurement method. If the target is stationary, the remaining DC-offset is constant. Otherwise, it is likely to change over time as the target position varies. If the DC-offset changes slowly, it can be said that it is constant for some short time interval. With this assumption, also called quasi-stationary, (3) can be written as

4 $$\begin{array}{rcl}{r}_{i}(t) & = & {A}_{0,i}\,\cos \,(4\pi {\lambda }^{-1}{d}_{i}+4\pi {\lambda }^{-1}x(t)+{\phi }_{0}+{\rm{\Delta }}\varphi (t))\\  &  & +\,j{A}_{0,i}\,\sin \,(4\pi {\lambda }^{-1}{d}_{i}+4\pi {\lambda }^{-1}x(t)+{\phi }_{0}+{\rm{\Delta }}\varphi (t))\\  &  & +\,D{C}_{I,i}+jD{C}_{Q,i}+w(t),\end{array}$$

where 0 ≤ *t* < *T*, *i* = 0, 1, 2, …, *r*_*i*_(*t*) = *r*(*t* − *iT*), and *T* is the quasi-stationary time interval. This assumption is reasonable in the case of the vital signal measurement where the chest displacement frequency is much slower than the radar sampling rate.

We assume that the phase noise is small Δ*ϕ*(*t*) ≈ 0, then (4) can be simply written as

5 $$\begin{array}{rcl}{r}_{i}(t) & \simeq  & {A}_{0,i}\,\cos \,(4\pi {\lambda }^{-1}{d}_{i}+4\pi {\lambda }^{-1}x(t)+{\phi }_{0})\\  &  & +\,j{A}_{0,i}\,\sin \,(4\pi {\lambda }^{-1}{d}_{i}+4\pi {\lambda }^{-1}x(t)+{\phi }_{0})\\  &  & +\,D{C}_{I,i}+jD{C}_{Q,i}+w(t).\end{array}$$

**Figure 2:**
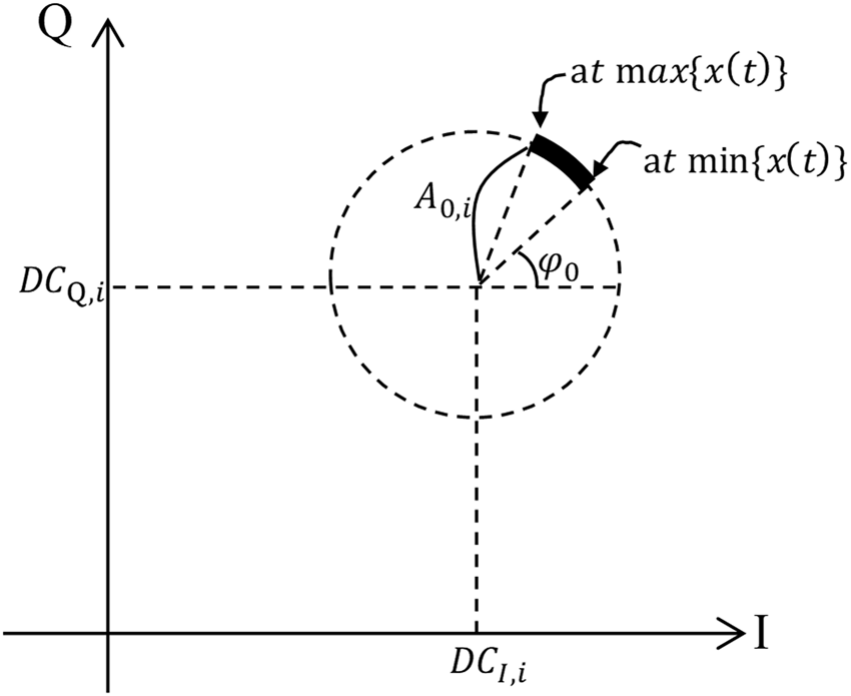
Complex plot representation of *r*_*i*_(*t*): *r*_*i*_(*t*) due to the displacement *x*(*t*) is located in the thick arc.

When *SNR* is high, (5) can be plotted as a part of a circle in a complex plane as shown in Fig. 2. *d* gives no information in the complex plane because *d* is shown as multiple rotations tracing on the dotted circle. To simplify the explanation, we eliminate the *d* term in this section as

6 $${r}_{i}(t)\simeq {A}_{0,i}\,\cos \,(4\pi {\lambda }^{-1}{x}_{i}+{\phi }_{0})+j{A}_{0,i}\,\sin \,(4\pi {\lambda }^{-1}{x}_{i}+{\phi }_{0})+D{C}_{I,i}+jD{C}_{Q,i}+w(t).$$

After the estimation of the center of the circle (*DC*_*I*_, *DC*_*Q*_) the displacement *x*_*i*_ can be obtained by linear and non-linear demodulation methods^4,26,27^. With the arctangent demodulation^4^, the displacement *x*(*t*) is easily obtained as

7 $$\arctan (\frac{\sin \,(4\pi {\lambda }^{-1}x(t)+{\phi }_{0})}{\cos \,(4\pi {\lambda }^{-1}x(t)+{\phi }_{0})})=4\pi {\lambda }^{-1}x(t)+{\phi }_{0}.$$

Various methods have been studied to estimate the center of circle^5,16,17,28^. In Park *et al*.^16^, *r*_*i*_(*t*) was first rotated parallel to the Q-axis. After the rotation DC-offset exists only on the I-axis, i.e., $(\hat{k},0)$. Then, $\hat{k}$ for two different time *t*_1_ and *t*_2_ is obtained by the following heuristic estimator

8 $$\hat{k}=median[\frac{{|{r}_{i}({t}_{1})|}^{2}-{|{r}_{i}({t}_{2})|}^{2}}{2Re\{{r}_{i}({t}_{1})-{r}_{i}({t}_{2})\}}].$$

The matrix of eigenvectors of the covariance matrix of *r*_*i*_(*t*) transforms the arc of *r*_*i*_(*t*) parallel to Q-axis, where the arc refers to the deviation of *r*_*i*_(*t*). This method is called Park method.

Unlike Park *et al*.^16^, Zakrzewsk *et al*.^5^ used a traditional circle fitting method, namely “geometric fit”, to estimate the center of circle. The DC-offset is obtained by minimizing the following function based on the geometric distance

9 $$E=\sum _{n=1}^{N}\,{(\sqrt{{(real({r}_{i}({t}_{n}))-D{C}_{I,i})}^{2}+{(imag({r}_{i}({t}_{n}))-D{C}_{Q,i})}^{2}}-R)}^{2},$$

where *R* is the radius of the fitting circle. Zakrzewsk *et al*.^5^ showed that (9) can be minimized by the classical Gauss-Newton method with the Levenberg–Marquardt (LM) correction factor λ. This method is called LM method. Details of this method are presented in Chernov *et al*.^28^. The Park method and LM method were compared in Zakrzewsk *et al*.^5^ through simulations and human respiration test. Computation time of the Park method is longer than the LM method because the eigenvalue calculation of covariance matrix in the Park method has higher computational complexity than the iterative operations in the LM method. In Zakrzewsk *et al*.^5^, it was shown that the LM method performs better than the Park method when the displacement is simple sinusoidal or complicated respiration.

#### Quadrature Frequency-Group Radar

**Figure 3:**
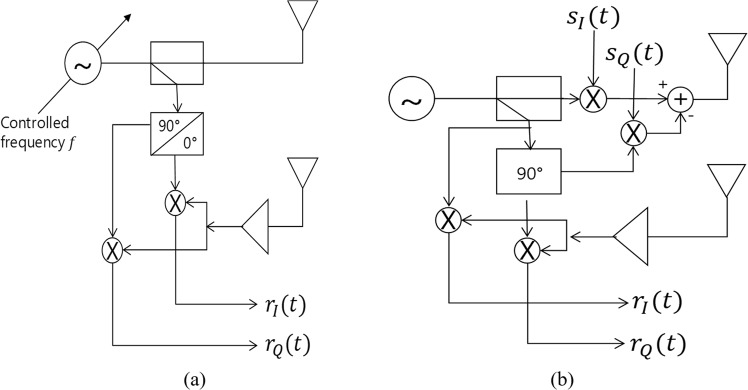
The QFG radar architecture: (**a**) SFCW-type and (**b**) OFDM-type.

For the QFG radar, the oscillator frequency *f* should be variable. To achieve this, two widely used architectures, step-frequency CW (SFCW) modulation and orthogonal-frequency-division-multiplexing (OFDM) modulation as depicted in Fig. 3(a,b), can be used^29–34^. The baseband signal in Fig. 3(a) is written as

10 $$\begin{array}{rcl}{r}_{i}(t,{f}_{m}) & = & {A}_{0,i}(m)\,\cos \,(4\pi {c}^{-1}{f}_{m}p(t)+{\phi }_{0})\\  &  & +\,j\cdot {A}_{0,i}(m)\,\sin \,(4\pi {c}^{-1}{f}_{m}p(t)+{\phi }_{0})\\  &  & +\,D{C}_{I,i}+jD{C}_{Q,i}+w(t),\end{array}$$

where *f*_*m*_ is the discrete frequency value that is controlled by the oscillator, *m* = 0, …, *M* − 1, and *p*(*t*) = *d*_*i*_ + *x*(*t*). If the frequency sweep time is sufficiently fast, the displacement *x*(*t*) is considered to be constant for the sweep time. Therefore, (10) can be written as

11 $$\begin{array}{rcl}{r}_{i}(n,{f}_{m}) & = & {A}_{0,i}(m)\,\cos \,(4\pi {c}^{-1}{f}_{m}p(n)+{\phi }_{0})\\  &  & +\,j\cdot {A}_{0,i}(m)\,\sin \,(4\pi {c}^{-1}{f}_{m}p(n)+{\phi }_{0})\\  &  & +\,D{C}_{I,i}+jD{C}_{Q,i}+w(n),\end{array}$$

where *p*_*i*_(*n*) = *d*_*i*_ + *x*(*n*), and *w*(*n*) is considered as a 2D Gaussian distribution with zero mean. The noise variance is expressed as signal-to-noise ratio (*SNR*). (11) is the baseband signal of the QFG radar. The QFG radar can also be implemented as in Fig. 3(b), where the frequency of the oscillator is fixed. But the transmitted baseband signal, *s*_*I*_(*t*) and *s*_*Q*_(*t*), is an OFDM symbol that is multiplexed by multiple frequency signals where each signal is orthogonal to the others. By multiplying the received baseband signal by the transmitted symbol which is a well-known method in OFDM communication systems, the same signal as (11) can be obtained. Thus, the QFG radar can be implemented through the above SFCW and OFDM architectures. We use the SFCW architecture in this paper.

**Figure 4:**
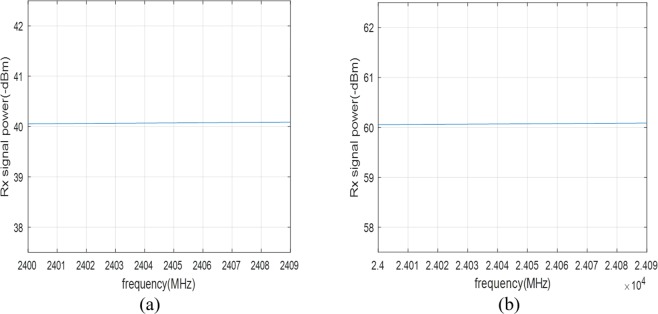
Received signal power (Transmission signal power is assumed to 1 mW): (**a**) 24 *GHz* band and (**b**) 2.4 *GHz* band.

The set of frequencies, i.e., F = {*f*_0_, *f*_1_, …, *f*_*M*−1_} = {*f*_*c*_, *f*_*c*_ + Δ*f*, …, *f*_*c*_ + (*M* − 1) · Δ*f*} lies within a frequency band, where *f*_*c*_ is the carrier frequency of F, and Δ*f* is the adjacent frequency difference. The popular carrier frequency bands of radar systems are 2.4 *GHz*, 10 *GHz*, 24 *GHz*, and etc. For these bands, a set of frequencies can be collected whose received signal power values do not vary considerably over F. For example, Fig. 4(a) shows the received signal power of *f*_*c*_ = 24 *GHz* and Δ*f* = 30 *MHz*, and Fig. 4(b) shows the received signal power of *f*_*c*_ = 2.4 *GHz* and Δ*f* = 3 *MHz*, where the transmitted signal power is assumed to be 1 *mW* and the path loss of the transmitted signal is considered as the free-space path loss equation^35^ as follows

12 $$FSPL(p(n),{f}_{m})=20lo{g}_{10}(p(n){f}_{m})-147.55,$$

where *p*(*n*) is set to 1 *m*. In the figures, the variance of the received signal powers is less than 1 *dB*. This kind of frequency set F is called a frequency group in this paper. In this case, it can be assumed that *A*_0,*i*_(*m*) is independent of *m*. Thus (11) can be written as

13 $${q}_{i}(n,{f}_{m})={A}_{0,i}exp[j4\pi {c}^{-1}{f}_{m}p(n)+{\phi }_{0}]+(D{C}_{I,i}+jD{C}_{Q,i})+w(n)$$

**Figure 5:**
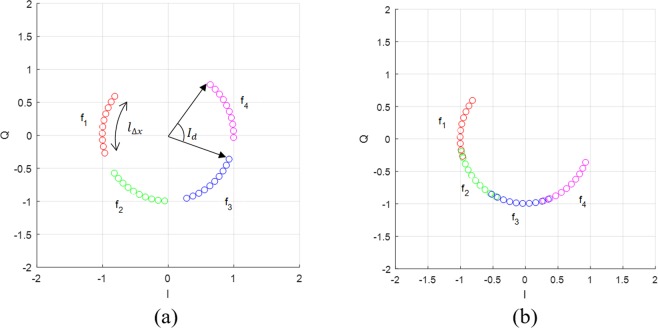
Multiple arcs of the QFG radar for Δ*x* = 1 *mm*: (**a**) Δ*f* = 30 *MHz* and (**b**) Δ*f* = 20 *MHz*.

(13) is hold in the rest of this section, and the effect of the assumption (13) on the center estimation performance will be discussed later. Then, (13) is an arc function of *f*_*m*_ and *p*(*n*) on a circle with its center and radius is (*DC*_*I*,*I*_, *DC*_*Q*,*i*_, *A*_*0*,i_). In this section, we assume that the dc-offset (*DC*_*I*,*I*_, *DC*_*Q*,*i*_) is zero for ease of explanation. For *d*_*i*_ = 30 *cm* and 0 < *x*(*n*) < 1 *mm*, the baseband signal of the QFG radar produces four arcs in complex plane as shown in Fig. 5(a), in which frequency group is set as {*f*_*c*_ = 24 *GHz*, Δ*f* = 30 *MHz*, *M* = 3}. If Δ*f* is reduced to 20 *MHz*, the arcs are overlapped as in Fig. 5(b). This shows that the arc can be extended by the QFG radar that makes up Δ*f* and *M*.

**Figure 6:**
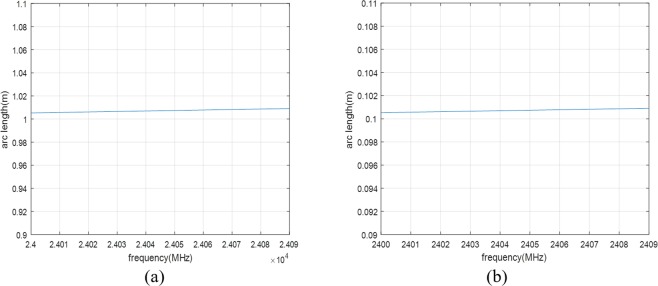
Arc length ${\rm{\Delta }}{\vartheta }_{x}(m)$ is almost constant with respect to *m*: (**a**) 24 *GHz* band and (**b**) 2.4 *GHz* band.

The interval of two adjacent arcs as shown in Fig. 6(a) can be obtained by

14 $${I}_{d}=\angle {q}_{i}(n,{f}_{m+1})-\angle {q}_{i}(n,{f}_{m})=4\pi {c}^{-1}{\rm{\Delta }}f\cdot d,$$

where $\angle {q}_{i}(n,{f}_{m})$ is the angle of *q*_*i*_(*n*, *f*_*m*_), and *I*_*d*_ depends on *d* and Δ*f*. Let Δ*x* the absolute displacement of *x*(*n*), and the arc length due to the displacement Δ*x* at *f*_*m*_ as shown in Fig. 6(a) is written as

15 $${l}_{{\rm{\Delta }}x}(m)={A}_{0,i}4\pi {c}^{-1}({f}_{c}+m{\rm{\Delta }}f){\rm{\Delta }}x,$$

As a rule of a thumb, Δ*x* is less than the order of 10^−3^, *f*_*c*_ is greater than the order of 10^9^, and Δ*f* is greater than the order of 10^6^. Then, *l*_Δ*x*_(*m*) is almost constant over *m* = 0, …, *M* − 1. Figure 6 shows *l*_Δ*x*_(*m*) of the arcs shown in Fig. 5, where the average length of the arcs is 10^3^ times greater than the maximum difference length of the arcs. The design of the QFG radar is simplified by approximating *l*_Δ*x*_(*m*) to *l*_Δ*x*_, thus (15) is written as

16 $${l}_{{\rm{\Delta }}x}={A}_{0,i}4\pi {c}^{-1}{f}_{c}{\rm{\Delta }}x,$$

**Figure 7:**
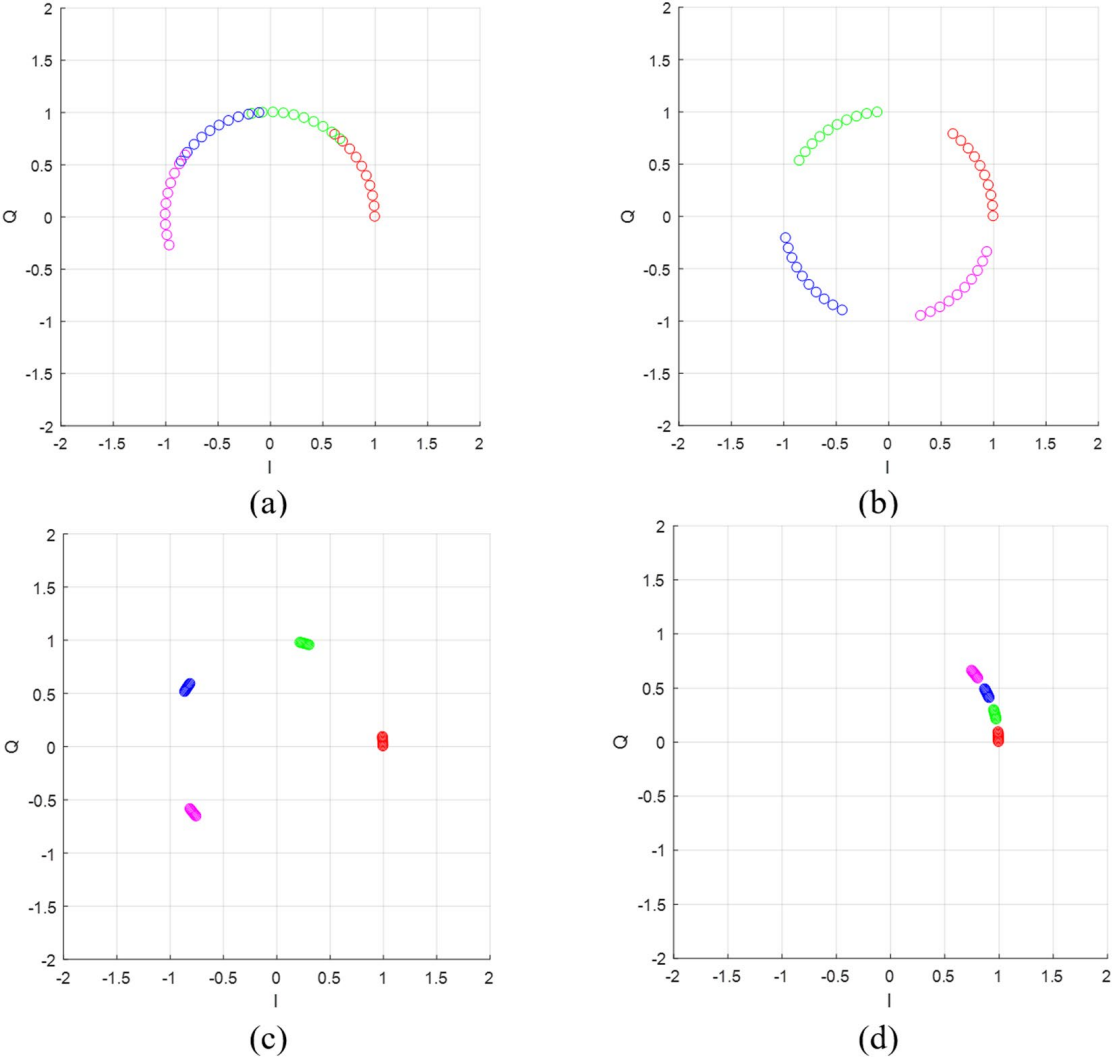
Examples at *f*_*c*_ = 24 *GHz*, *M* = 4: (**a**) Δ*f* = 30 *MHz* for Δ*x* = 1 *mm*, (**b**) Δ*f* = 40 *MHz* for Δ*x* = 1 *mm* (**c**) Δ*f* = 30 *MHz* for Δ*x* = 0.1 *mm*, and (**d**) Δ*f* = 5 *MHz* for Δ*x* = 0.1 *mm*.

Using (14) and (16), we can determine the parameter of the QFG radar such as *f*_*c*_, Δ*f*, and *M* according to a given Δ*x* and *d*. For example, consider that Δ*x* = 0.1 ~1 *mm* and *d* = 30 *cm* (mostly, respiration and heartbeat radars have this range of Δ*x*. First, we should determine *f*_*c*_ of which wavelength is greater than Δ*x*. For Δ*x* = 1 *mm*, we selected *f*_*c*_ = 24 *GHz* of which wavelength is sufficiently greater than the maximum Δ*x* = 1 *mm*. Assuming that *A*_0,*i*_ = 1, *l*_Δ*x*_ ≈ 1 and then we can determine Δ*f* using (14) by considering how much we separate the adjacent arcs. By selecting Δ*f* = 30 *MHz*, *I*_*d*_ ≈ 0.377 and the adjacent arcs are located as shown in Fig. 7(a), where arcs are properly placed and slightly overlapped. Because *l*_Δ*x*_ ≈ 1 is small, we need to extend it using *M*. For *M* = 4, the arc length is extended to almodt four times as shouwn in Fig. 7(a). If Δ*f* = 40 *MHz*, total length of the arcs are longer than Δ*f* = 30 *MHz* but the arcs are discontinuously placed as depicted in Fig. 7(b). For small displacement such as Δ*x* = 0.1 *mm*, Δ*f* should be set small and bigger *M* if you want to get continuously placed long arcs. By reducing Δ*f* to 5 *MHz*, the arcs are continuously placed in contrast to Δ*f* = 30 *MHz*, which are shown in Fig. 7(c,d), respectively. These arcs are directly related to the performance of their center estimation algorithms as will be presented in the following sections.

#### Center Estimation of the QFG Radar

**Figure 8:**
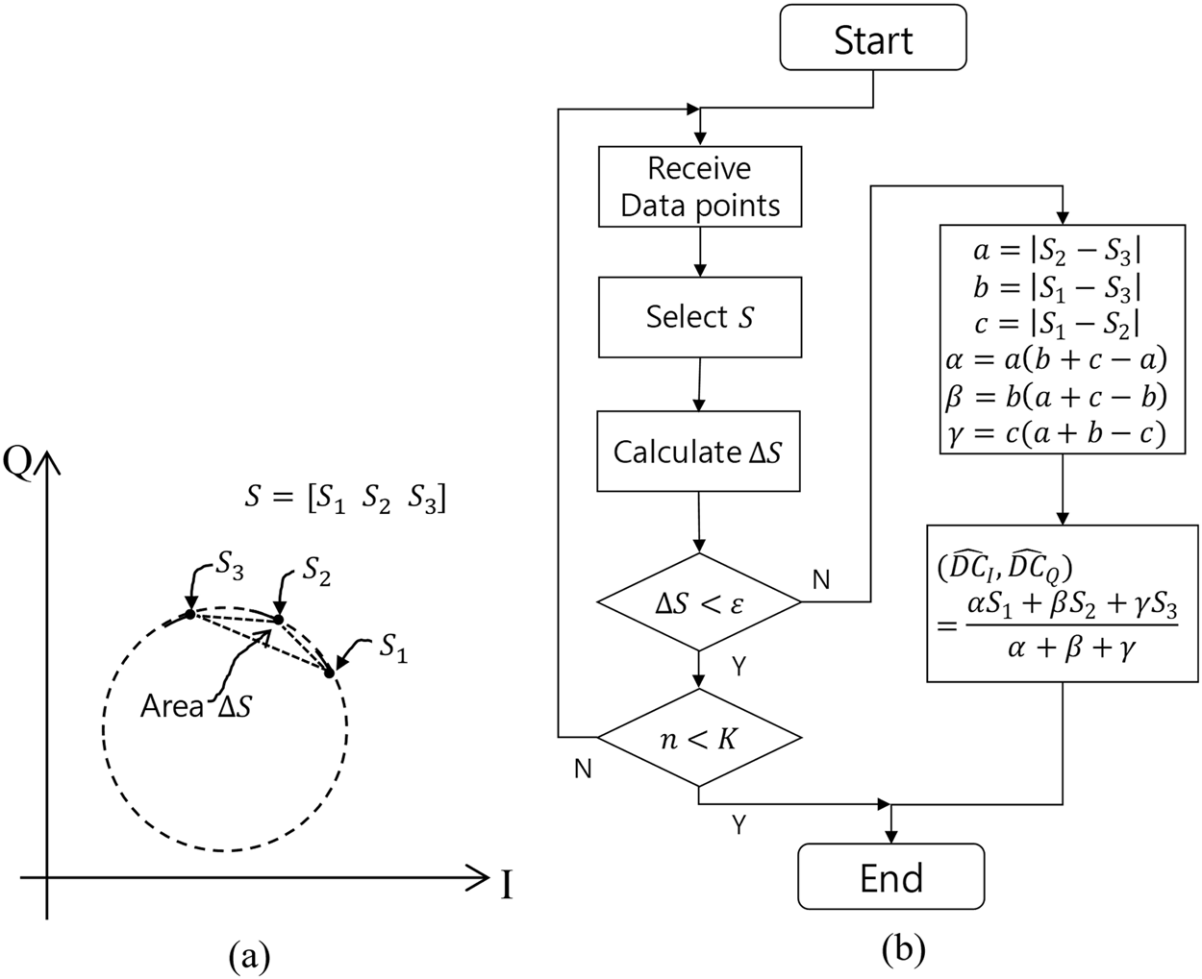
Circumcenter method: (**a**) If area ${\rm{\Delta }}S > \epsilon$, the $({\widehat{DC}}_{I},{\widehat{DC}}_{Q})$ is the circumcenter of the triangle *S*. (**b**) Flowchart.

We starts from (11) with *M* = 3. Three algorithms are presented to estimate the center of (11) for the QFG radar: the circumcenter method (computationally simple)^36^, the Pratt method (algebraic fitting)^37^, and the LM method (geometric fitting)^38^. The circumcenter calculation method is as follows. Three points are selected as $S=[{y}_{i}(n,{f}_{0})\,{y}_{i}(n,{f}_{1})\,{y}_{i}(n,{f}_{2})]$. Then, the area of *S*, Δ*S*, is calculated. If ${\rm{\Delta }}S > \epsilon$, the circumcenter can be calculated as

17 $$({\widehat{DC}}_{I},{\widehat{DC}}_{Q})=\frac{\alpha {S}_{1}+\beta {S}_{2}+\gamma {S}_{3}}{\alpha +\beta +\gamma },$$

where $\epsilon$ is a small value, e.g., 10^−9^, *α* = *a*(*b* + *c* − *a*), *β* = *b*(*a* + *c* − *b*), *γ* = *c*(*a* + *b* − *c*), *a* = |*S*_2_ − *S*_3_|, *b* = |*S*_1_ − *S*_3_|, and *c* = |*S*_1_ − *S*_2_|. If ${\rm{\Delta }}S < \epsilon$, another *S* should be taken for other *n* until ${\rm{\Delta }}S > \epsilon$. If there is no *S* that satisfies ${\rm{\Delta }}S > \epsilon$, the method fails. If Δ*f* is carefully designed considering *f*_*c*_, *d*, and Δ*x*, the method would not fail. This method is summarized in Fig. 8.

The Pratt method is algebraic circle fitting, which is a non-iterative procedure. Thus, the method is computationally efficient compared to the iterative geometric fitting method such as LM method. The algebraic fitting is the minimization of a circle polynomial:

18 $${ {\mathcal F} }_{ab}(A,B,C,D)=\sum _{j=0}^{J-1}\,\sum _{m=0}^{M-1}\,{z}^{2}(j,m),$$

where $z(j,m)=A{|{y}_{i}(j,{f}_{m})|}^{2}+B\cdot Re\{{y}_{i}(j,{f}_{m})\}+C\cdot Im\{{y}_{i}(j,{f}_{m})\}+D$ is called the circle polynomial or the *algebraic distance*. The Pratt method^37^ is the gradient weighted algebraic fitting that minimizes

19 $${ {\mathcal F} }_{Pratt}(A,B,C,D)=\sum _{j=0}^{J-1}\,\sum _{m=0}^{M-1}\,\frac{{z}^{2}(j,m)}{{\Vert \nabla z(j,m)\Vert }^{2}},$$

where ∇ is the function gradient operator. By approximating *z*(*j*, *m*) ≈ 0, (19) can be simplified as

20 $${ {\mathcal F} }_{Pratt}(A,B,C,D)=\sum _{j=0}^{J-1}\,\sum _{m=0}^{M-1}\,\frac{{z}^{2}(j,m)}{{B}^{2}+{C}^{2}-4AC},$$

Minimization of (20) is equivalent to the minimization of (19) with the constraint *B*^2^ + *C*^2^ − 4*AC* = 1^37^. With the Lagrange multiplier *η*, the minimization can be written as

21 $${{\rm{G}}}^{T}({{\rm{X}}}^{T}X){\rm{G}}-\eta ({{\rm{G}}}^{T}H{\rm{G}}-1),$$

where G = [*A B C D*]^*T*^,

$${\rm{H}}=[\begin{array}{cccc}0 & 0 & 0 & -2\\ 0 & 1 & 0 & 0\\ 0 & 0 & 1 & 0\\ -2 & 0 & 0 & 0\end{array}],$$

$${\rm{X}}=[\begin{array}{c}{\rm{Y}}(0)\\ \vdots \\ {\rm{Y}}(J-1)\end{array}],$$

and

$${\rm{Y}}({\rm{n}})=[\begin{array}{cccc}{|{y}_{i}(n,{f}_{0})|}^{2} & Re\{{y}_{i}(n,{f}_{0})\} & Im\{{y}_{i}(n,{f}_{0})\} & 1\\ \vdots  & \vdots  & \vdots  & \vdots \\ {|{y}_{i}(n,{f}_{M-1})|}^{2} & Re\{{y}_{i}(n,{f}_{M-1})\} & Im\{{y}_{i}(n,{f}_{M-1})\} & 1\end{array}].$$

(21) is differentiated with A as follows:

22 $${{\rm{H}}}^{-1}({{\rm{X}}}^{T}X){\rm{G}}-\eta {\rm{G}}=0,$$

**Figure 9:**
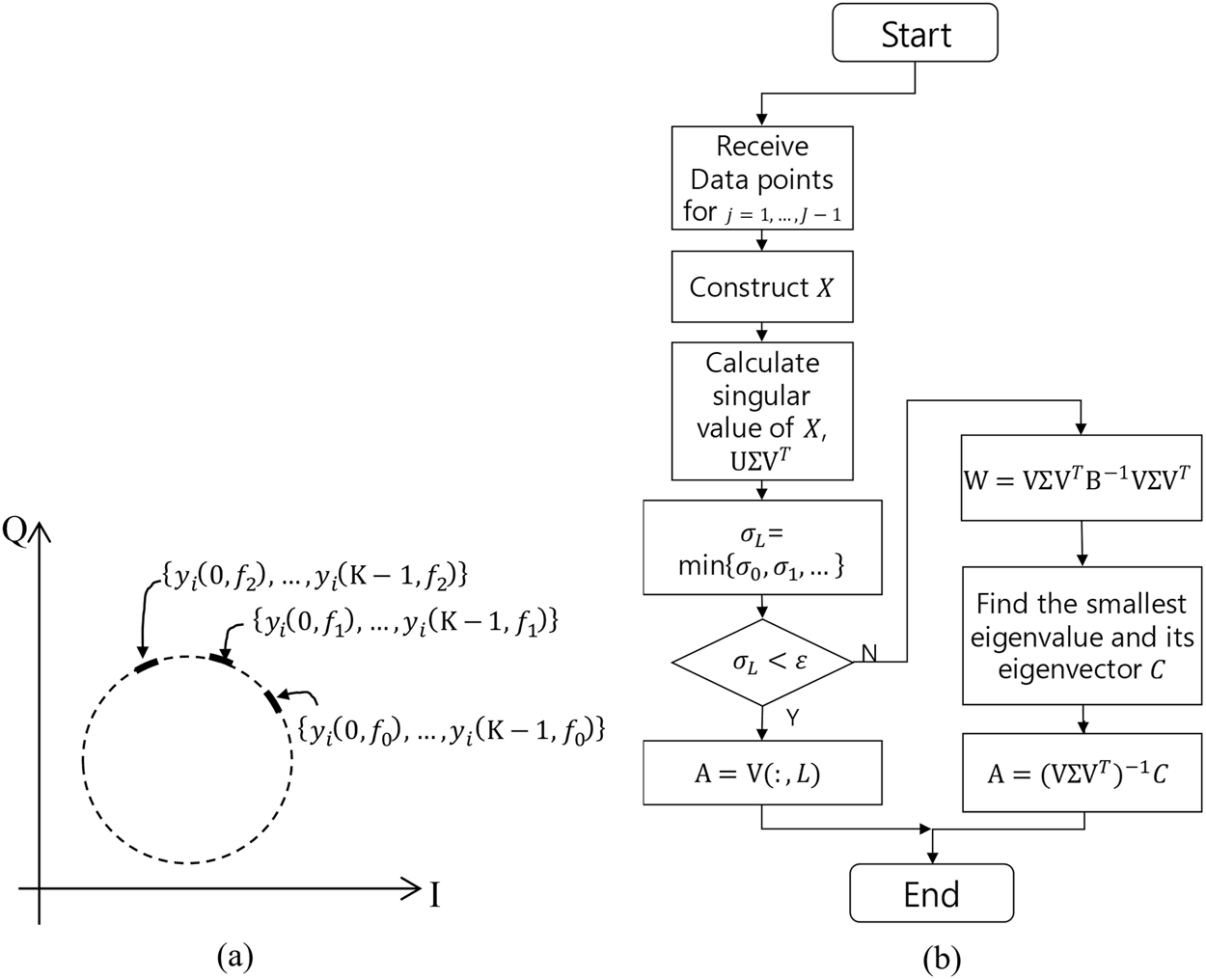
Pratt method: (**a**) Gathered data points and (**b**) Flowchart.

Hence (22) is the (*η*, G)-problem of the matrix H^−1^(X^*T*^ *X*), where *η* is the smallest non-negative eigenvalue and A is its eigenvector. In this paper, we solve the problem using the numerically stable singular value decomposition (SVD) of X, X = U∑V^*T*^. After SVD, compute W = V∑V^*T*^H^−1^V∑V^*T*^. Then, find the smallest eigenvalue and its eigenvector *C*. Finally, we obtain G = (V∑V^*T*^)^−1^*C*. In the singular case where the smallest positive singular value of X is less than a small-valued tolerance, G is just the right eigenvector of the smallest singular value. This method is summarized in Fig. 9.

The above circumcenter method and the Pratt method are non-iterative fitting method. Thus, they prefer largely distributed arcs data in the beginning of the calculation procedure. The QFG radar is adequate for providing such lengthy arcs with only a small displacement. On the other hand, the QCW radar having a short arc with a small displacement should use computationally complex fitting method such as geometric iterative fitting method. This kind of complex method is not recommended for the QFG radar, but is used in this paper for comparison of algorithms. In this paper the LM method is used for the comparison: the LM method is the most popular geometric fitting method^38^ and the detailed description can be found in Zakrzewski *et al*.^5^, Chernov *et al*.^28^, and Gander *et al*.^38^.

## Results

### Simulation Results

**Figure 10:**
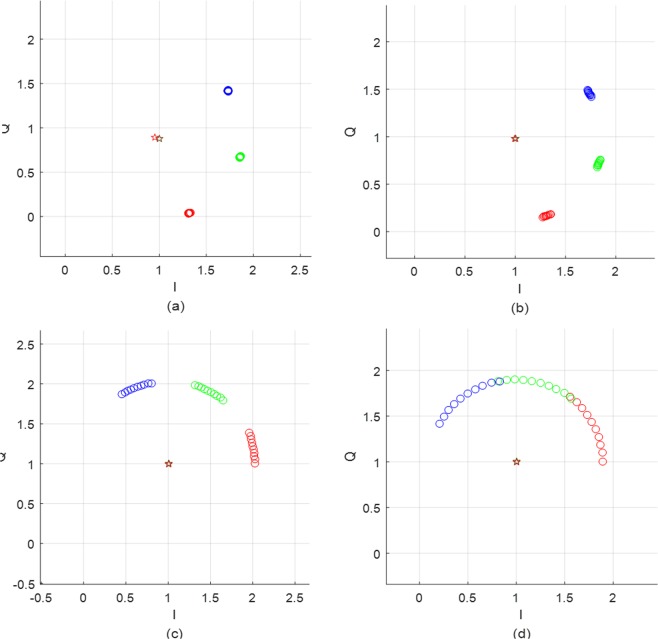
Arcs of the QFG radar for different *f*_*c*_ (Real center of the arcs are C (1, 1)): (**a**) *f*_*c*_ = 400 *MHz* (**b**) *f*_*c*_ = 2.4 *GHz* (**c**) *f*_*c*_ = 10 *GHz*, and (**d**) *f*_*c*_ = 24 *GHz*.

**Figure 11:**
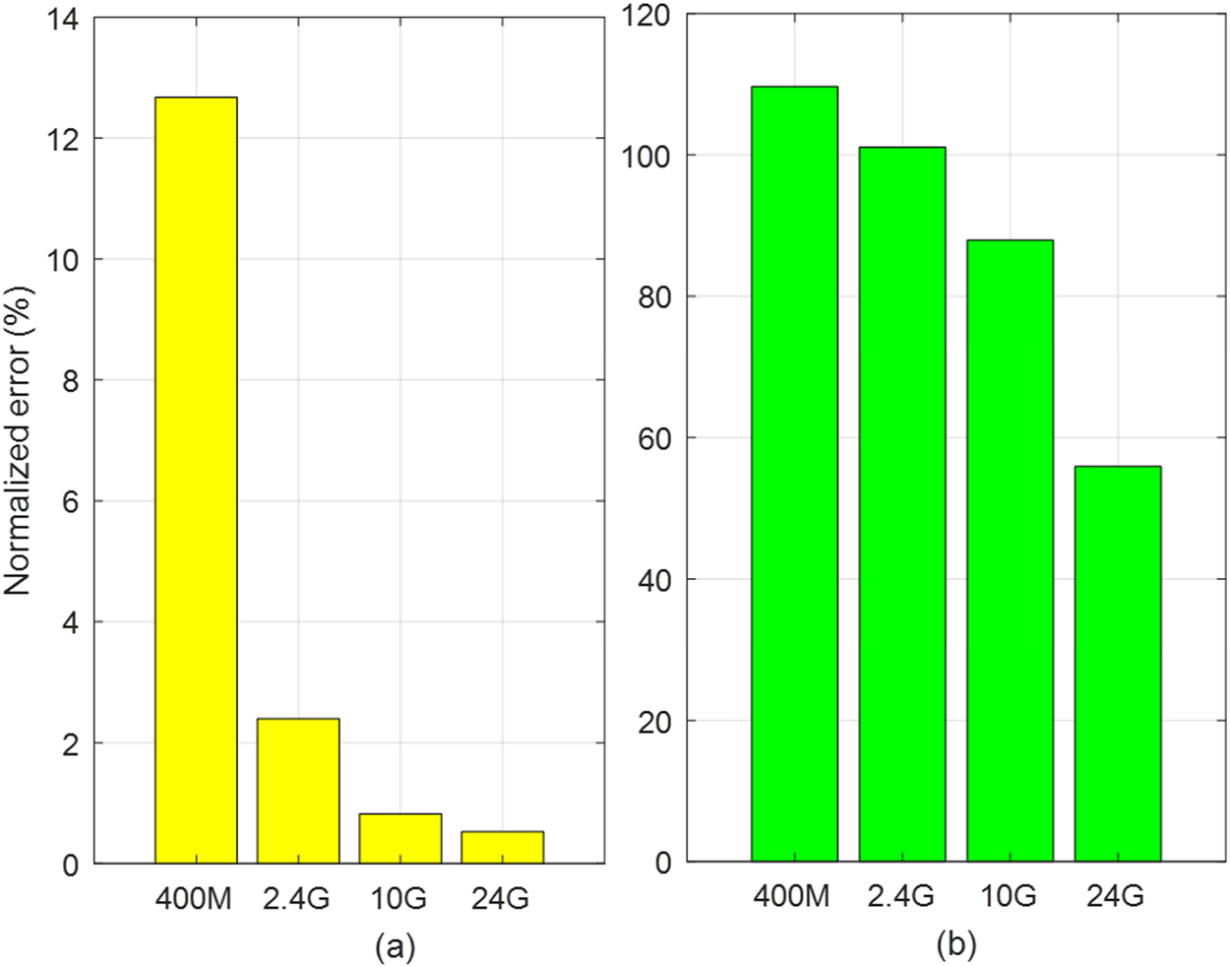
Normalized error for different carrier frequency bands for Δ*f* = 20 *MHz*: (**a**) the QFG radar and (**b**) the QCW radar.

In the simulations, the center estimation performance is mainly discussed. To do this, we first define a normalized estimation error, the similar definition is used in Gao *et al*.^21^:

23 $$Error=\frac{\Vert (D{C}_{I},D{C}_{Q})-({\widehat{DC}}_{I},{\widehat{DC}}_{Q})\Vert }{R}\times 100\,( \% ),$$

where (*DC*_*I*_, *DC*_*Q*_) is the real DC-offset, *R* is the radius of the real circle, and $({\widehat{DC}}_{I},{\widehat{DC}}_{Q})$ is the estimated DC-offset. First, we compare the QFG radar and QCW radar the following parameter settings; Δ*f* = 20 *MHz* and *M* = 3. Target is located at *d* = 30 *cm*, its movement is assumed to be sinusoidal displacement with Δ*x* = 1 *mm*, and the baseband *SNR* is set to 40 *dB*. The real DC-offset is (*DC*_*I*_, *DC*_*Q*_) = (1, 1). After the IQ-imbalance correction as described in previous section, the baseband signals of the QFG radar are shown in Fig. 10 for four different bands *f*_*c*_ = 400 *MHz*, 2.4 *GHz*, 10 *GHz*, 24 *GHz*. In the four complex planes in Fig. 10, star marks indicate the estimated DC-offset $({\widehat{DC}}_{I},{\widehat{DC}}_{Q})$; three estimation results of circumcenter, Pratt, and LM methods are placed as star marks in each complex plane. We define the arc possession in percentage as the total arc distribution over one wavelength. For the QCW radar, the arc possession for the four bands in Fig. 10 are 0.13%, 0.8%, 3.3%, and 8%, respectively. The QFG radar extends the arc possession to 27.5%, 31.5%, 46.7%, and 42.7% of the corresponding *f*_*c*_ although some extensions are discontinuous. This difference about the arc possession has a direct impact on the center estimation performance of the two radars. Figure 11 compares the normalized error of the QFG radar with QCW radar using the circumcenter method. Figure 11 shows that the error is reduced by the arc extension regardless of the arc continuity. The QCW radar shows significant performance degradation for all *f*_*c*_, but the QFG radar has much better performance. For *fc* ≥ 2.4 *GHz*, the error of the QFG radar is less than 3%.

**Figure 12:**
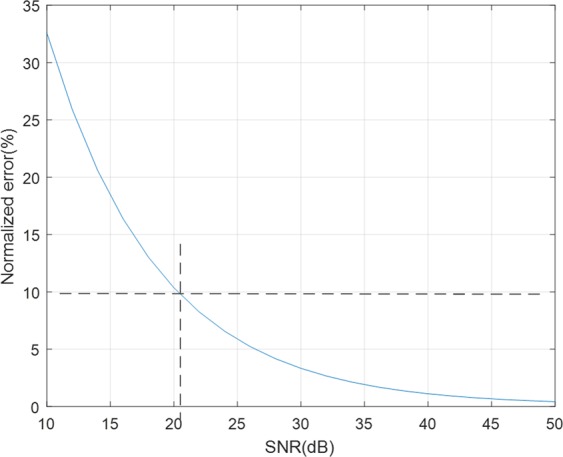
The effect of *SNR* on the error performance of circumcenter method.

**Figure 13:**
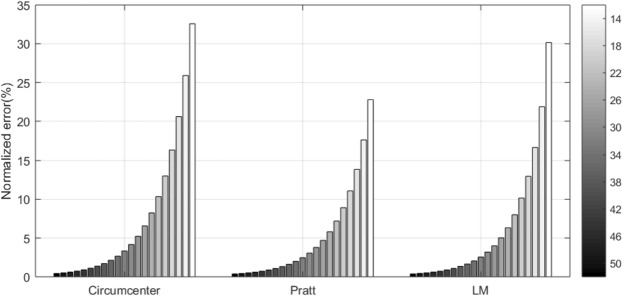
Performance comparison of three estimation methods according to *SNR* (Note:Arc possession is 42.7%).

Matlab R2017a is used for these simulations. 100 times of experiments are performed and calculated the average for each simulation. The following simulation shows how the normalized error is affected by *SNR*. It can be said that the higher *fc*, more sensitive the radar is to *SNR*. For *fc* = 24 *GHz*, the normalized error for *SNR* from 12 *dB* to 50 *dB* is shown in Fig. 12, in which the circumcenter method is used. To obtain the error less than 10%, at least *SNR* should be 21 *dB*. Figure 13 shows that the error is slightly lowered by using the Pratt and LM methods. The pure white and black bars indicate the errors for *SNR* = 10 *dB* and 50* dB*, respectively. At high *SNR*, the LM method is similar error rate to the Pratt method. On the other hand, at low *SNR*, the Pratt method is slightly better than the LM method.

**Figure 14:**
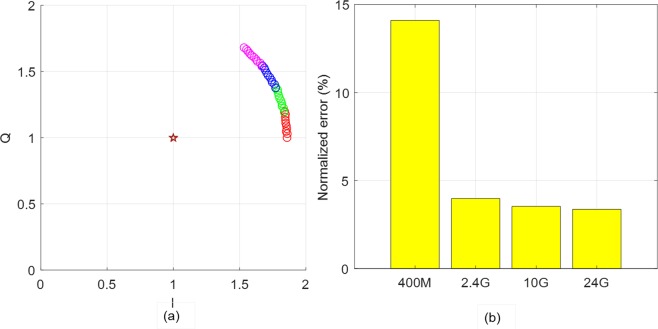
The QFG radar for Δ*f* = 3 *MHz*: (**a**) arcs and (**b**) normalized error for different carrier frequency bands.

In practice, smaller Δ*f* is preferred because the phase-locked loop (PLL), which is the frequency changing device, is fast and more stable for small frequency change. The reduced arc possession due to the small Δ*f* can be increased by large *M*. Thus, Δ*f* and *M* is a design factor when a hardware specification is given. For example, when Δ*f* = 3 *MHz*, Δ*x* = 0.5 *mm*, and *M* = 4, the arc possessions of the QFG radar for above the four *f*_*c*_s are about 20%, where the arcs for *f*_*c*_ = 24 *GHz* is depicted in Fig. 14(a). In this case, the normalized error performance of the circumcenter method at *SNR*=30 dB is as depicted in Fig. 14(b). Compared with Fig. 10, reduced arc possession due to some overlapped sections affects the center estimation performance. When preferring small Δ*f*, *M* is required to be properly set according to Δ*f* and Δ*x*.

## Experimental Results

**Figure 15:**
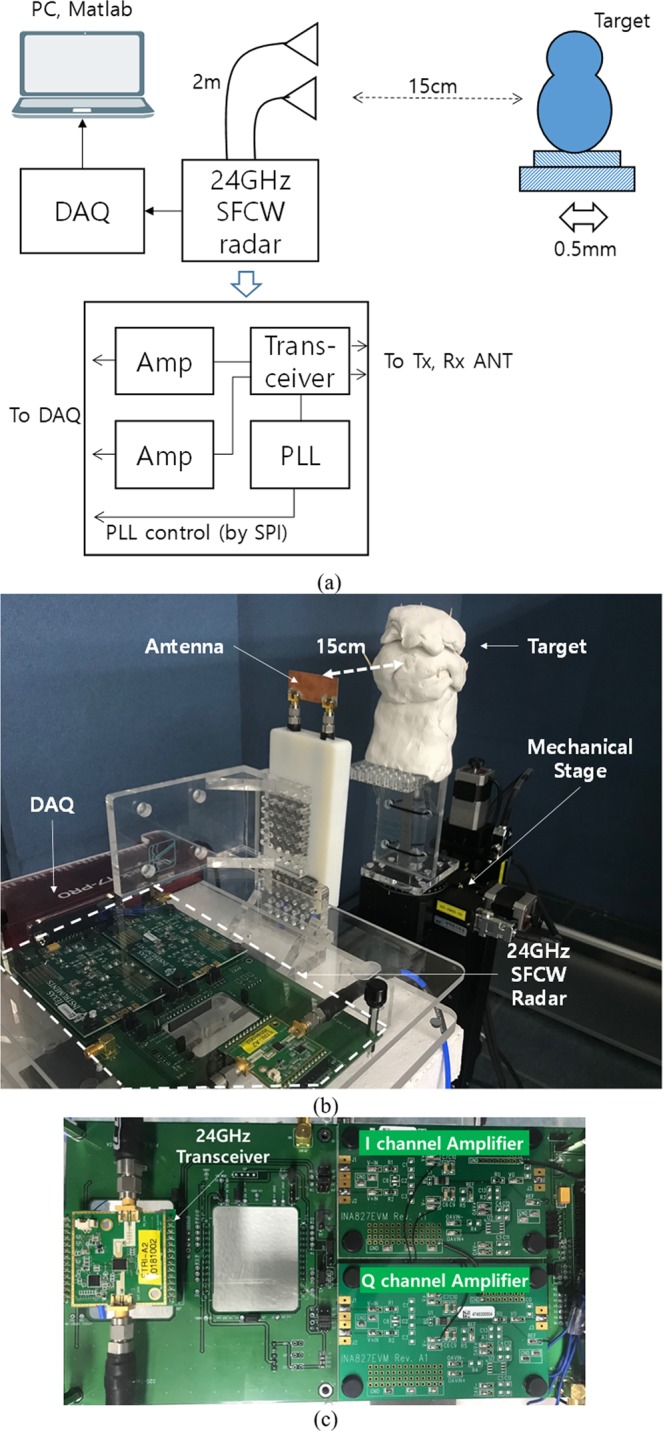
(**a**) Measurement setup (**b**) Photograph (**c**) The SFCW-type radar system.

**Figure 16:**
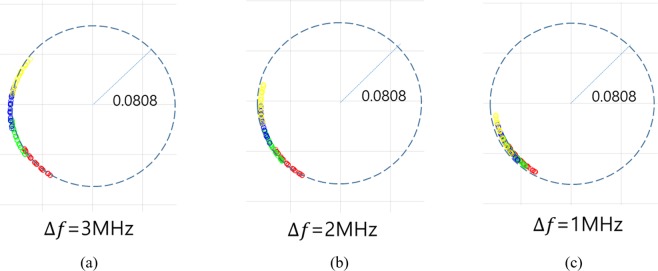
Arcs of the QFG radar in the experiment. Red, green, blue, and yellow arcs are for four frequencies elements. The dashed circle with the center at (0, 0) is the ground truth.

**Figure 17:**
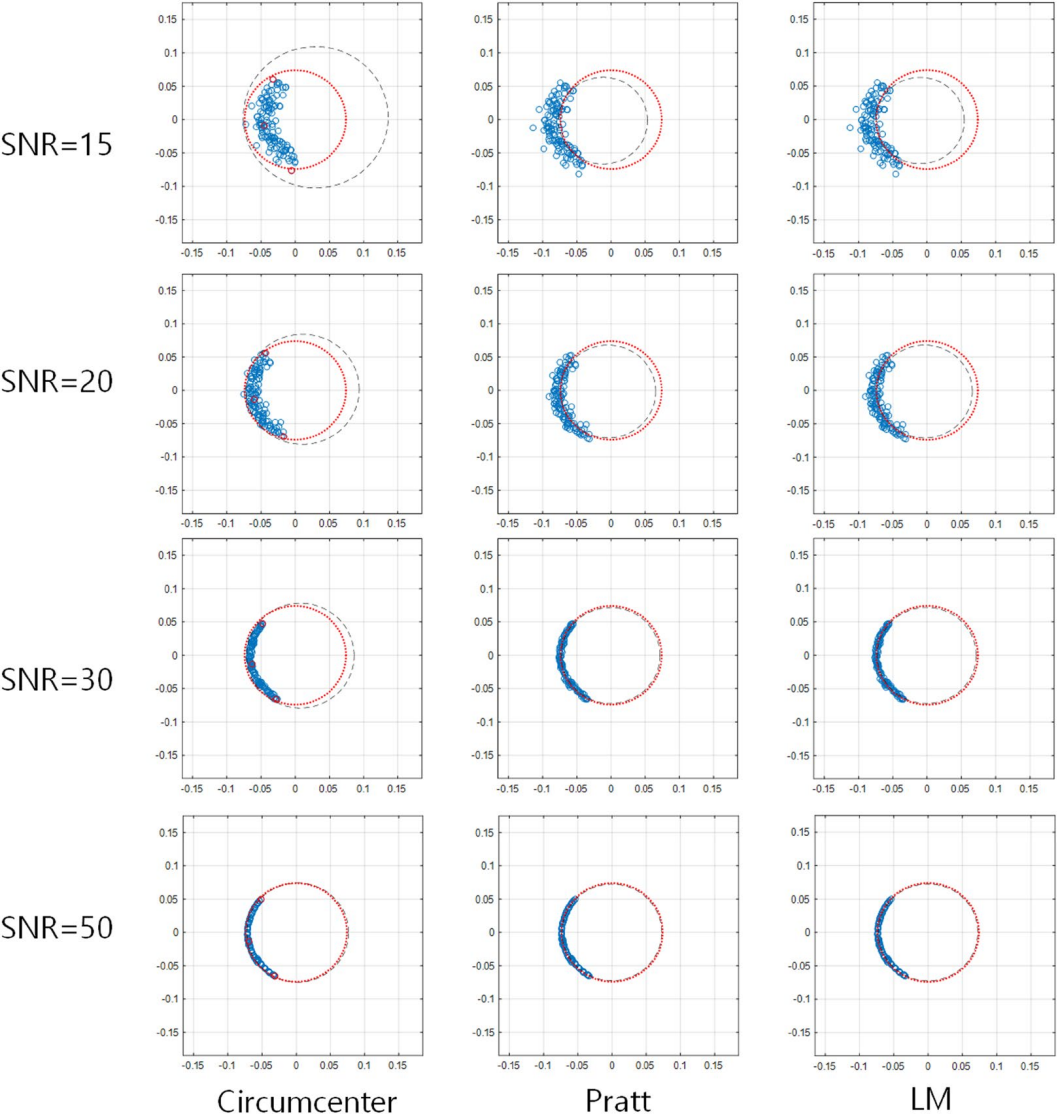
Fitting results on noisy dataset for three algorithms. Red dotted circle is the ground truth and the black dashed circle is the fitted result. (Note:Arc possession is 20.2%).

**Figure 18:**
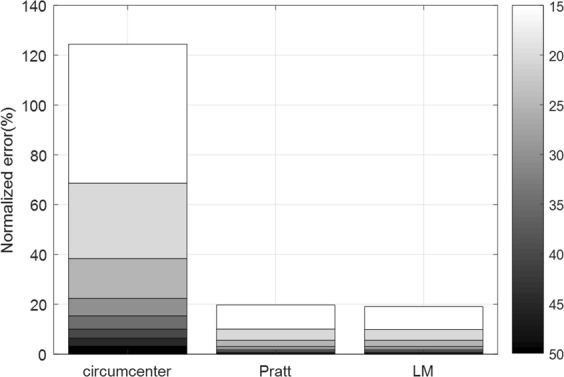
Performance comparison of three estimation methods for the QFG radar over various *SNRs* (Note:Arc possession is 20.2%).

The feasibility of the proposed QFG radar is experimentally evaluated in the measurement setup as shown in Fig. 15. The transceiver of the SFCW-type radar is an Infineon BGT24LTR operating at 24 GHz with an output power of 1 dBm. The PLL is a Texas Instrument LMX2491, and the I/Q amplifier is a Texas Instrument INA827 of which gain is 7 *dB*. Antenna has 5 dBi gain and 43° half-power beamwidth, which is placed 15 *cm* away from the target. The mechanical stage is used to create displacement of 0.5 *mm*. The target having uneven surface is mounted on the stage. Its height and width are 35 *cm* and 25 *cm*. The unevenness is good for making environmental impairment easily. The cables between the radar and antenna are Pasternack PE350, of which each length is 2 *m* and dielectric constant is 2.30. The PC acquires the received signals I and Q through a Labjack T7-Pro DAQ. Matlab R2017a is used for controlling the radar, running center estimation algorithms, and adding white Gaussian noise. For experiments, we have three frequency groups ${{\rm{F}}}_{1},{{\rm{F}}}_{2},\,{\rm{and}}\,{{\rm{F}}}_{3}$ according to Δ*f* = 1 *MHz*, 2 *MHz*, and 3 *MHz*, respectively. Each frequency group has four frequency elements and their *f*_*c*_ is 24.000 *GHz*. In the first experiment, we compared the equations about arc length (16) and arc interval (14) with the experiment results at the high *SNR* = 50 *dB*. The IQ-imbalance was corrected using (3), and The Pratt method was used to estimate the DC-offset. Figure 16 shows the resultant arcs of the QFG radar in this experiment, where four arcs are displayed in each chart and the radius of the arcs are 0.808. The measured arc length and interval values are summarized in Table 1, in which the average values are almost same as equations (16) and (14). As discussed earlier in previous section, the arc interval is reduced when Δ*f* is small. One thing to note here is that the cable helps to increase the arc interval when the distance between the radar and the target is closely placed. Actually, when Δ*f* = 1 *MHz* the cables themselves increase the arc interval by about 7.3 degrees. The total arc possession of this experiment is summarized in Table 2(a). The QFG radar gives the arc possession of 20.2% at Δ*f* = 3 *MHz* compared to only 5.1% for the QCW radar, and their normalized center estimation errors are summarized in Table 2(b). The performance of the QFG radar at Δ*f* = 3 *MHz* is experimented over *SNR* from 15 *dB*. Fitted circles on some *SNR*s are depicted in Fig. 17. As *SNR* is decreased, the deviation from the ground truth circle is increased for the three fitting algorithms. Performance of the normalized center estimation error for all *SNR* ranges are shown in Fig. 18. For sufficiently high *SNR*, *SNR* > *30* *dB* in this experiment, the three algorithms showed feasible results. The Pratt and LM methods were shown to be feasible in all *SNR* ranges. Thus, when the available *SNR* is known, the appropriate fitting algorithm can be selected. After performing the center estimation, it is easy to calculate the displacement. For Δ*f* = 3 *MHz* and *SNR* = 30 *dB*, the calculations for Δ*x* = 0.3 *mm*, 0.5 *mm*, 1 *mm* are performed and shown in Table 3, where 50 vibrations are measured and averaged for each Δ*x*. The relative errors of the displacement calculation are 2.3%, 1.4%, and 0.8%, respectively. These results are comparable with the results using CW Doppler radar^39^, where the results^39^ used the pre-calibration step to get the DC-offset using sufficiently long arc while the QFG radar uses no pre-calibration step.

**Table 1:** Arc length and interval values in the experiment shown in Fig. 16.

Arc length *l*_Δ*x*_				Arc interval *I*_d_ (radian)			
Δ*f*(MHz)	3	2	1	Δ*f*(MHz)	3	2	1
m = 0	0.039	0.04	0.037	m = 0	23.7	16.9	8.23
m = 1	0.038	0.0396	0.037	m = 1	22.1	15	7.84
m = 2	0.042	0.0395	0.0406	m = 2	23.9	15.2	7.27
m = 3	0.041	0.0407	0.0418	Average	23.3	15.7	7.78
Average	0.04	0.04	0.0392	Eq. (14)	23.46	15.64	7.82
Eq. (16)		0.04		—			

Note that arc length is dimensionless and the radius of the arcs are 0.808.

**Table 2:** (a) Arc Possession of the experiment shown in Fig. 16, (b) Normalized Erro.

(a)			
Δ*f*(MHz)	3	$2$	$1$
Possession(%)	20.2	13.9	7.26
**(b)**			
**Method**		**Normalized error (%)**	
		**QFG radar**	**QCW radar**
Circumcenter		3.54	16.4
Pratt		0.16	16.1
LM		0.15	16.1

**Table 3:** Comparison of displacement measurement accuracy (QFG radar: Δ*f* = 3 *MHz*, and SNR = 30 dB, CW radar: SNR is not mentioned in^39^ but it can be assumed that the SNR is sufficiently high SNR enough)).

Δ*x* (mm)	QFG radar w/o pre-calibration step			CW radar using pre-calibration step with sufficiently long arc^39^		
	Avg. (mm)	Avg. error (um)	Rel. error (%)	Avg. (mm)	Avg. error (um)	Rel. error (%)
0.3	0.307	7	2.3	0.307	7	2.33
0.5	0.494	6	1.4	0.507	7	1.34
1	1.008	8	0.8	0.992	8	0.78

Simulation and experiment results show that the circumcenter method has inferior performance compared to the Pratt and LM methods, especially for low *SNR*s, because the circumcenter method estimates the center by using only three points *S* among the available data points, whereas the other two methods use all the data points. However, for high *SNR*s, the circumcenter method is advantageous compared to the other methods because the circumcenter method is computationally simple. In this paper, we consider the time required for MATLAB to compute the center estimation methods by using the profile function in MATLAB, where MATLAB was run on the Intel i7-7500U processor at the clock frequency 2.7 GHz. For various *SNR*s, the execution times of the methods are shown in Table 4, in which each time value refers to one execution time. The execution times are measured with 100 iterations and averaged for each simulation. For each method, 40 complex data points were put. For LM method, iteration threshold was set to 10^−6^, and the maximum iteration number was limited to 50. The circumcenter method has the smallest computation time of around 0.16 ms. The Pratt and LM methods require twice and four times the amount of computation time that the circumcenter method does, respectively. For the LM method, the lower the *SNR*, the longer the computation time because the number of iterations increases.

**Table 4:** Computational complexity.

*SNR*(dB)	Circumcenter (ms)	Pratt (ms)	LM (ms)
10	0.165	0.32	0.615
20	0.165	0.32	0.585
30	0.16	0.315	0.58
40	0.16	0.31	0.575
50	0.16	0.31	0.565

Execution times are measured with 100 iterations and averaged for each simulation^40–44^.

## Discussion

The micro-Doppler radar is a promising technique for small vibrational displacement such as non-contact vital signal sensing. It can be used as sleep monitoring, driver drowsiness/fatigue detection, buried survivor searching, and other human motion classifying applications. The QFG radar is useful for estimating the center of a circle where enough arcs are hard to be obtained. The QFG effectively extends arc length with such a small displacement which helps improving the performance of the abovementioned center estimation algorithms. Some simulation results have been shown for various carrier frequency bands with different Δ*f*, and *M*. Experimental results have been shown using 24 *GHz* SFCW-type radar system with the target of 0.5 *mm* displacement. Both of the simulation and experimental results have shown that the QFG radar outperforms the QCW radar. Of course, the parameters of the QFG have to be properly designed for the given environment, which was discussed through some simulations. The QFG radar can serve as a DC-offset tracking method for real-time applications under the uncontrollable environment because the arc is sufficiently provided and the curve fitting algorithms can be implemented using the off-the-shelf embedded processors.
